# If the shoe fits… should you just wear it? A complete calcaneal stress fracture in a female recreational runner

**DOI:** 10.17159/2078-516X/2020/v32i1a8522

**Published:** 2020-01-01

**Authors:** J W Burger, R de Villiers, W Derman

**Affiliations:** 1 Institute of Sport and Exercise Medicine, Division of Orthopaedic Surgery, Department of Surgical Sciences, Faculty of Medicine and Health Sciences, Stellenbosch University, South Africa; 2 Department of Psychiatry and Mental Health, Faculty of Health Sciences, University of Cape Town, South Africa; 3 Winelands Radiology, Institute of Orthopaedics and Rheumatology, Stellenbosch University, South Africa; 4 IOC Research Centre, South Africa

**Keywords:** bone stress, injury, running, footwear, minimalist

## Abstract

Shoe choice by runners may follow trends related to purported generalised benefits rather than following an individual risk-benefit analysis. The benefits and risks related to minimalist footwear for running has been a much debated topic. The authors report a case of a complete calcaneal stress fracture in an otherwise healthy female recreational runner in the first three weeks following her conversion from a traditional cushioned running shoe to a minimalist type of running shoe. Clinicians should be aware of the potential added bone stress with reduced cushioning and the potential risks in transitioning to new footwear.

## Case report

### History

A healthy 43-year-old female recreational runner presented to sports practitioners with severe right heel pain. The reported pain was on the lateral side of her foot, radiating up her calf when she walked. There was also mild foot swelling on activity. She was no longer able to run and had consulted multiple practitioners without receiving a diagnosis.

She had been running for the past 12 years with no previous history of severe injury. During the preceding six months, she averaged 50–60 km of running per week and had completed 17 races of between five km and 21 km. She had no recent increases in training volume or intensity.

On further inquiry, it was revealed that she had recently changed from a traditional cushioned running shoe to a more minimalist type of running shoe. This new, lighter pair of running shoes had a two mm heel-toe drop, with a stack height of 23 mm at the heel. Within three weeks of this conversion, she had completed a 21 km and a five km race. The day after completion of the five km race, she struggled to bear weight on her right foot.

There was no noteworthy medical, psychiatric, or surgical history and no current menstrual abnormalities.

### Physical examination

On examination, she was physically well, with a Body Mass Index of 19.24. There was unilateral, generalised tenderness over her right calcaneus, with no tenderness noted on calf palpation. No further abnormalities were found on physical examination.

The initial differential diagnosis included bone stress injury of the calcaneus, plantar fascial injury, nerve entrapment, and retrocalcaneal bursitis.

### Special investigations

X-rays of the foot revealed an oblique fracture of the calcaneus, with surrounding sclerosis (Fig. 1a).

Magnetic Resonance Imaging (MRI) of the hind foot was conducted to exclude any other concurrent pathology. The MRI showed a linear STIR-hypointense line extending from the posterosuperior to the anteroinferior aspect of the calcaneus, indicating a complete fracture of the right calcaneus, with associated bone marrow oedema (Fig. 1b).

A DEXA scan revealed normal bone mineral density (BMD) and blood tests showed that the patient’s vitamin D levels were within the normal range. Further tests of liver function, thyroid function, ferritin, and infective markers were all normal.

### Diagnosis and management

A final diagnosis of a complete fracture of the right calcaneus was made. Initial management consisted of non-weight bearing in a controlled ankle motion walking boot with crutches, followed by partial weight bearing in a controlled ankle motion walking boot. A graded return-to-sport programme was followed successfully without incident. This programme included swimming to maintain cardiorespiratory fitness, with a stepwise addition of more weight-bearing exercise through the use of an elliptical, walking, and ultimately, running. She was running again after four months and completed her first 10 km race six months following her injury.

Currently, running is her main form of exercise, and she covers 40–50 km per week. She has had no further bone stress injuries after returning to traditional cushioned shoes.

## Discussion

Bone stress injuries are common sport-related injuries in athletes and can represent up to 20% of injuries that present to sports physicians.^[1]^ These injuries may occur in normal bone in response to excessive loads or repeated load cycles, or in weakened bone as a result of normal loads. With repeated stress and inadequate recovery, local osteoclastic activity exceeds the osteoblastic capacity and subsequent loading causes microfractures in the trabecular bone, before eventually causing breaks in the cortical bone if the stress pattern continues.^[1]^

Many bones in the lower limb sustain these injuries and their individual incidence varies according to sport, although the tibia, fibula, and metatarsals are some of the most frequent sites.^[1]^ Although bone stress injuries of the calcaneus are well-described as a differential of heel pain, this case is unusual as the bone stress injury of the patient’s calcaneus developed into a significant complete facture over a short period of time, and in the absence of any abnormality of bone density or other medical concerns.

There are many recognised extrinsic and intrinsic risk factors for a bone stress injury that have been identified, but the exact role that footwear plays in the pathogenesis of these injuries has been contentious.^[2]^ Typically, a bone stress injury follows sudden or rapid increases of load stress cycles during weight-bearing training.^[1]^ Other recognised risk factors for a bone stress injury include female gender, menstrual dysfunction, low bone mineral density, low caloric intake and, most importantly, combinations of these factors.^[1]^

Since the 2000s, there has been an increase in the popularity of minimalist running shoes, owing to the initial hypothesis that they may reduce injury or improve running economy.^[3]^ However, neither traditional cushioned shoes nor minimalist shoes have been shown to reduce overall injury rates.^[2]^ Minimalist shoes may merely change the type of injuries that are seen, rather than the overall frequency of injuries.

Minimalist running shoes aim to mimic the biomechanics of barefoot running, strengthen musculature, and reduce stress on the lower limbs, while still providing protection from the surfaces on which one runs.^[3]^ There is some evidence that minimalist shoes may improve running economy, but these changes come with increased loading of the ankle, Achilles tendon, and the metatarsophalangeal joint.^[2]^ It is not yet clear whether this change in running economy is due to non-rearfoot striking or simply the reduced shoe weight.^[2,4]^ The vast majority of runners (between 75–95%) are rear-foot strikers and almost half of recreational runners may continue to rear-foot strike in minimalist shoes.^[5]^ With reduced cushioning at the heel in minimalist shoes, this could potentially place these runners at an increased risk for stress fractures. However, there is currently insufficient evidence to recommend changing a healthy runner’s strike pattern. In fact, it could be potentially harmful to change loading patterns, with limited benefits.^[4]^ It is often in the transition periods between changing shoes or foot-strike where there is the potential for injury.^[2,6]^

## Conclusion

Transitioning between shoe types or running form could potentially cause harm to the runner through injury. After 12 years of running without significant injury, this recreational runner developed a bone stress injury in the three weeks following a change in her shoe type, which progressed to a complete fracture of the right calcaneus. Clinicians should exercise caution when dealing with uninjured, healthy athletes who wish to change their shoes and should be able to counsel them on the risks and benefits of transitioning.

## Figures and Tables

**Fig. 1a:**
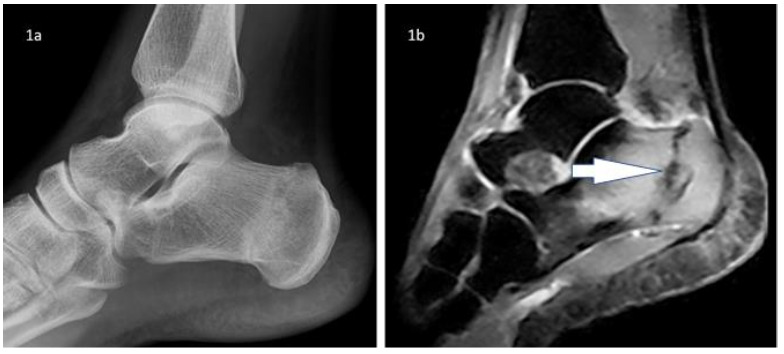
Lateral X-ray showing oblique stress fracture of the calcaneus with surrounding sclerosis.

**Fig. 1b:**
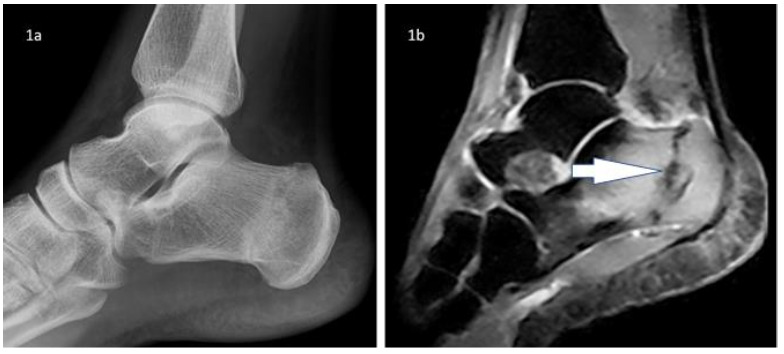
Sagittal STIR ankle showing hypointense fracture line with surrounding bone marrow oedema.
